# Respiratory-resolved five-dimensional flow cardiovascular magnetic resonance : In-vivo validation and respiratory-dependent flow changes in healthy volunteers and patients with congenital heart disease

**DOI:** 10.1016/j.jocmr.2024.101077

**Published:** 2024-08-02

**Authors:** Elizabeth K. Weiss, Justin Baraboo, Cynthia K. Rigsby, Joshua D. Robinson, Liliana Ma, Mariana B.L. Falcão, Christopher W. Roy, Matthias Stuber, Michael Markl

**Affiliations:** a Department of Radiology, Northwestern University, Chicago, Illinois, USA; b Department of Biomedical Engineering, Northwestern University, Evanston, Illinois, USA; c Department of Medical Imaging, Ann & Robert Lurie Children’s Hospital, Chicago, Illinois, USA; d Department of Cardiology, Ann & Robert Lurie Children’s Hospital, Chicago, Illinois, USA; e Department of Diagnostic and Interventional Radiology, Lausanne University Hospital (CHUV) and University of Lausanne (UNIL), Lausanne, Switzerland

**Keywords:** 5D flow CMR, Respiratory-resolved flow, Real-time phase-contrast MRI, Congenital heart disease, Single ventricle disease

## Abstract

**Background:**

This study aimed to validate respiratory-resolved five-dimensional (5D) flow cardiovascular magnetic resonance (CMR) against real-time two-dimensional (2D) phase-contrast MRI, assess the impact of number of respiratory states, and measure the impact of respiration on hemodynamics in congenital heart disease (CHD) patients.

**Methods:**

Respiratory-resolved 5D flow MRI-derived net and peak flow measurements were compared to real-time 2D phase-contrast MRI-derived measurements in 10 healthy volunteers. Pulmonary-to-systemic flow ratios (Qp:Qs) were measured in 19 CHD patients and aortopulmonary collateral burden was measured in 5 Fontan patients. Additionally, the impact of number of respiratory states on measured respiratory-driven net flow changes was investigated in 10 healthy volunteers and 19 CHD patients (shunt physiology, n = 11, single ventricle disease [SVD], n = 8).

**Results:**

There was good agreement between 5D flow MRI and real-time 2D phase-contrast–derived net and peak flow. Respiratory-driven changes had a good correlation (rho = 0.64, p < 0.001). In healthy volunteers, fewer than four respiratory states reduced measured respiratory-driven flow changes in veins (5.2 mL/cycle, p < 0.001) and arteries (1.7 mL/cycle, p = 0.05). Respiration drove substantial venous net flow changes in SVD (64% change) and shunt patients (57% change). Respiration had significantly greater impact in SVD patients compared to shunt patients in the right and left pulmonary arteries (46% vs 15%, p = 0.003 and 59% vs 20%, p = 0.002). Qp:Qs varied by 37 ± 24% over respiration in SVD patients and 12 ± 20% in shunt patients. Aortopulmonary collateral burden varied by 118 ± 84% over respiration in Fontan patients. The smallest collateral burden was measured during active inspiration in all patients and the greatest burden was during active expiration in four of five patients. Reduced respiratory resolution blunted measured flow changes in the caval veins of shunt and SVD patients (p < 0.005).

**Conclusions:**

Respiratory-resolved 5D flow MRI measurements agree with real-time 2D phase contrast. Venous measurements are sensitive to number of respiratory states, whereas arterial measurements are more robust. Respiration has a substantial impact on caval vein flow, Qp:Qs, and collateral burden in CHD patients.

## Graphical abstract

**Figure:**
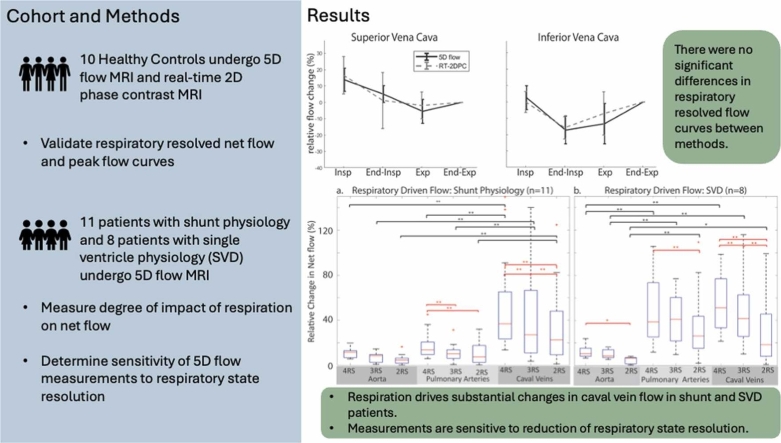

## 1 Background

Four-dimensional (4D) flow cardiovascular magnetic resonance (CMR) has shown promise for evaluating cardiac and vascular hemodynamics in pediatric and adult patients with cardiovascular abnormalities [1], [2], [3], [4]. In congenital heart disease (CHD), 4D flow CMR is particularly useful for its ability to assess hemodynamics in complex, three-dimensional anatomy resolved over the cardiac cycle with full volumetric coverage of the heart and thorax [5]. However, widespread adoption has been impeded by several limitations of the technique, including long and unpredictable scan times dependent on heart rate and respiration control efficiency, as well as cumbersome cardiac and respiratory gating methods [6], [7]. Free-running five-dimensional (5D) flow CMR [8], [9], [10], [11] was recently introduced as a novel technique that eliminates several drawbacks of 4D flow CMR. 5D flow CMR is based on continuous radial k-space sampling with a predictable scan time, using cardiac and respiratory self-gating, and compressed sensing reconstruction to enable high levels of scan acceleration. This method removes the need for electrocardiogram (ECG) lead placement and respiratory navigators, simplifying the acquisition process. Additionally, the combination of radial k-space sampling and inherent self-gating allows for the reconstruction of respiratory-resolved 5D flow CMR data which provides information about the impact of the respiratory state (RS) (e.g., inspiration vs expiration) on cardiovascular hemodynamics [8], [9], [10], [11].

It is well known that respiration drives hemodynamic changes. For instance, cardiac catheterization and real-time MRI studies have shown that left ventricular stroke volumes increase during expiration while right ventricular stroke volumes increase during inspiration [12], [13], [14]. More recently, MRI has been used to measure respiratory-driven flow changes. 5D flow MRI methods have been used to show respiratory-dependent net flow changes in the caval veins of patients with valve disease [8] and single ventricle physiology [11]. These studies highlight respiration as an important driver of hemodynamics in single ventricle physiology patients, where the pulmonary circulation is passively filled. However, the clinical impact of respiratory-resolved hemodynamics has yet to be investigated. Additionally, in patients with shunt physiology, where pulmonary and systemic circulations have a pathologic connection, respiration may be expected to play a role in hemodynamics [15], [16]. In particular, it is possible that the shunt volume, a metric used in determining treatment planning [17], [18], varies over the respiratory cycle. Due to the importance of hemodynamics in CHD management, it is important to better understand flow variation over respiration in the evaluation of both single ventricle disease (SVD) and shunt patients. Additionally, new physiologic information may be uncovered by measuring respiratory-driven flow dynamics which may be used to improve patient management.

While the 5D flow CMR method is promising, prior studies have several limitations in validating and characterizing 5D flow CMR. The self-gating respiratory signal derived from 5D flow CMR has not been compared against a ground truth and respiratory-driven changes in flow measured by 5D flow MRI have not yet been validated against a reference standard. Additionally, the optimal number of respiratory states has yet to be investigated.

The purpose of this study was to address these limitations. We aimed to (1) validate the respiratory self-gating signal from 5D flow CMR against a respiratory bellows signal, (2) validate respiratory-driven flow changes measured by 5D flow CMR against real-time 2D phase-contrast CMR (RT-2DPC CMR) as a reference, using ECG and independently acquired respiratory signal for physiologic gating, (3) assess the impact of respiration on the pulmonary-to-systemic flow ratio (Qp:Qs) in patients with shunt physiology and aortopulmonary collateral (APC) burden in patients with single ventricle physiology, and (4) investigate the impact of number of respiratory states on measured respiratory-driven flow changes in the arteries and veins in a cohort of healthy volunteers and patients with CHD.

## 2 Methods

### 2.1 Study cohort

A total of 10 healthy volunteers (39.7 ± 21.6 years, 20% female) were prospectively recruited for a research MRI exam. Volunteers were required to be 18 years or older. Exclusion criteria included a known history of heart disease and contraindications to CMR, such as metallic implants. Additionally, 19 pediatric patients with CHD (14.5 ± 9.1 years, 53% female, 32% under general anesthesia) undergoing clinically indicated, standard-of-care CMR studies were prospectively recruited for a 5D flow CMR add-on scan. Patients with SVD who were status-post Fontan procedure, status-post Glenn procedure, and those with an intracardiac or extracardiac shunt were included in the cohort. Exclusion criteria included contraindications to CMR. Two subgroups were defined: shunt physiology (n = 11) and SVD (n = 8). The shunt physiology group included intracardiac and extracardiac shunts. The SVD group included patients with a Glenn connection or total cavopulmonary connection. This study was Health Insurance Portability and Accountability Act compliant and institutional review board approved and all participants, or their legal guardians, provided written informed consent. As appropriate, minors were assented as well.

### 2.2 MR imaging: volunteer study

All volunteers underwent a single scanning session without contrast, including 5D flow MRI and RT-2DPC CMR. External ECG leads and abdominal respiratory bellows were placed on all volunteers at the beginning of the study. The 5D flow MRI data were acquired based on a previously reported pulse sequence [8]. Briefly, free-running data were continuously acquired with a radial phyllotaxis k-space trajectory for a set scan time of 8.5 minutes. Periodic superior-inferior readouts were used to retrospectively extract cardiac and respiratory motion signals for self-gating. For every five 4-point velocity-encoding (venc) readouts acquired, one superior-inferior readout was acquired. A total of 24,100 velocity encoding interleafs were acquired. The RT-2DPC MRI method was a Cartesian echo planar imaging (EPI) sequence with kt-generalized autocalibrating partially parallel acquisition (GRAPPA, R factor = 2, EPI factor = 11) accelerated reconstruction [19]. This method was used to assess respiratory-driven flow changes in a 2D acquisition. 2D imaging planes for RT-2DPC were placed at the following anatomic locations: ascending aorta (AAo), main pulmonary artery (MPA), inferior vena cava (IVC), and superior vena cava (SVC). Total scan time for RT-2DPC CMR was 38 seconds per 2D image location. Scan parameters for both sequences are summarized in Table 1. All scans were performed on a 1.5T MR system (Aera, Siemens Healthineers, Erlangen, Germany).

**Table 1:** Scan parameters for patient and volunteer studies.

Scan parameter	5D flow CMR (CHD patients)	5D flow CMR (volunteers)	RT-2DPC CMR
Spatial resolution (mm)	1.5–2.5 isotropic	2.5 isotropic	3.1 × 3.1 × 10
Temporal resolution (ms)	40 ms	40 ms	37.5 ms
FOV (mm)	144 × 144 × 144–240 × 240 × 240	240 × 240 × 240	350 × 400
TR (ms)	28–28.6	28–28.6	75.4
TE (ms)	2.9–3.0	2.9–3.0	4.9
Flip angle	25°	7°	7°
Contrast	ferumoxytol	none	none
Venc (m/s)	1.5–2.0	1.5–1.9	1.5–2.05
Reconstruction acceleration	Compressed sensing	Compressed sensing	kt-GRAPPA
Acquisition time	8.5 min	8.5 min	38 s

*5D* five-dimensional*, CMR* cardiovascular magnetic resonance*, RT-2DPC* real-time 2D phase-contrast*, FOV* field-of-view*, TE* echo time*, venc* velocity-encoding*, TR* repetion time, *CHD* congenital heart disease, kt-GRAPPA generalized autocalibrating partially parallel aquisitions*.*

### 2.3 CMR: congenital heart disease patients

All CHD patients underwent an 8.5 minute add-on 5D flow CMR at the end of their clinically indicated CMR study. Intravascular contrast agent (Feraheme, AMAG Pharmaceuticals, Waltham, Massachusetts) was previously administered for a clinically indicated sequence in all patients included for flow analysis. Scan parameters for this cohort are also summarized in Table 1. A total of 24,100 velocity encoding interleafs were acquired. All scans were performed on a 1.5T MR system (Aera, Siemens Healthineers, Erlangen, Germany).

### 2.4 5D flow CMR image reconstruction: cardiac and respiratory self-gating and compressed sensing

**Fig. 1:**
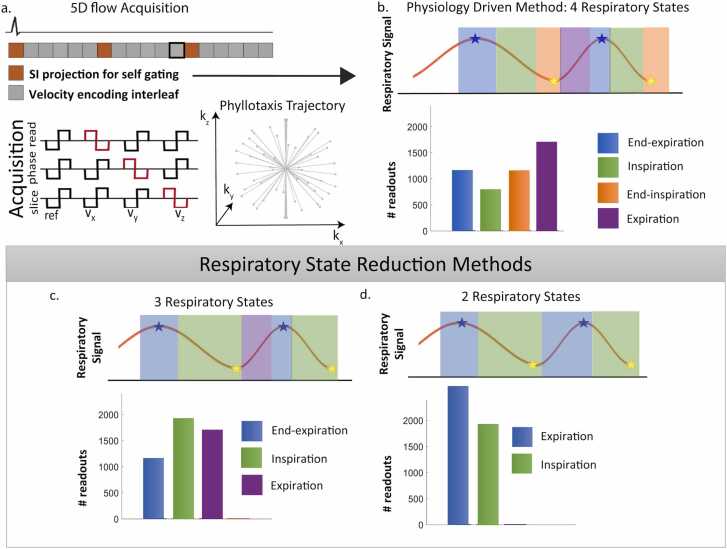
5D flow CMR pulse sequence diagram and radial phyllotaxis trajectory (a). A peak-finding algorithm is used to find inspiratory peaks (yellow stars) and expiratory peaks (blue stars) to perform breath-wise respiratory gating (b). Modifications to the method for three respiratory states (c) and two respiratory states (d) reconstructions. *5D* five-dimensional, *CMR* cardiovascular magnetic resonance, *SI* superior-inferior.

The respiratory signal was extracted and filtered from the self-gating superior-inferior readouts, as previously described [8], [20]. A peak-finding algorithm was used to determine the location of peak expiration and peak inspiration [21]. The signal was then gated on a breath-by-breath basis (Fig. 1a) and the 5D flow CMR raw data were reconstructed into four respiratory states. For each breath, defined as two consecutive expiratory peaks, the k-space data acquired within the top 25th percentile of signal magnitude were assigned to end-expiration. K-space data with the bottom 25th percentile of magnitude were assigned to end-inspiration. The k-space data acquired before end-inspiratory were assigned to inspiration, and the remaining k-space data to expiration. In all cases, 40 ms cardiac temporal resolution was used for retrospective cardiac gating. All reconstructions were completed using compressed sensing on a high-performance computational cluster (Two 40 GB Tesla A100 graphics processing units (GPUs), 52 central processing unit (CPU) cores, and 192 GB random access memory). Anonymized raw data were uploaded directly to the Northwestern University-managed cluster (Quest) for image reconstruction. Acceleration rates were calculated separately for each respiratory state based on the number of k-space lines in each bin.

To assess the impact of the number of respiratory states on the sensitivity of 5D flow CMR to detect respiration-driven flow changes, the same 5D flow CMR raw data were also reconstructed with two or three respiratory states. To define three respiratory states, end-inspiration and inspiration were combined into a single respiratory state (Fig. 1b). To define two respiratory states, end-expiration and expiration were also combined into a single respiratory state (Fig. 1c). All images were anonymized before storage and analysis.

### 2.5 Self-gating respiratory signal validation

The respiratory bellows signal and extracted respiratory self-gating signal were compared in all volunteers. Cross-correlation was used to optimally align the signals, as a time shift in self-gating signal compared to bellows signal has been previously reported [22].

### 2.6 5D flow CMR post-processing

Each of the 2–4 respiration-resolved 5D datasets were analyzed using commercial software (Circle 5.14.2 Cardiovascular, CVI, Calgary, Alberta, Canada), including preprocessing with eddy current correction and velocity anti-aliasing. 2D analysis planes were manually placed in the following locations for healthy volunteers: ascending aorta (AAo), descending aorta (DAo), main pulmonary artery (MPA), left and right pulmonary arteries (LPA, RPA), superior vena cava (SVC), and inferior vena cava (IVC). In CHD patients, 2D analysis planes were placed in the following locations: AAo, MPA, RPA, LPA, IVC, and SVC. In Fontan patients, the pulmonary vein net flows were also measured. Each vessel lumen was manually delineated for each time frame. Net flow and peak flow were measured from time-resolved flow curves. Pulmonary-to-systemic flow ratios were quantified in all patients. In shunt patients, systemic flow (Qs) was defined as aortic net flow, and pulmonary flow (Qp) was defined as the MPA net flow. The shunt volume (Qp-Qs), reported in liters per minute, was calculated using the heart rate. In patients status-post Glenn procedure, Qp was defined as LPA + RPA net flow and Qs was defined by aortic net flow. In Fontan patients, Qp was defined as the sum of pulmonary vein flow and Qs was defined as the sum of caval vein flow (IVC + SVC). APC burden was quantified in Fontan patients as the difference in aortic flow and systemic venous return (IVC + SVC).

### 2.7 Real-time 2D phase-contrast CMR image processing

**Fig. 2:**
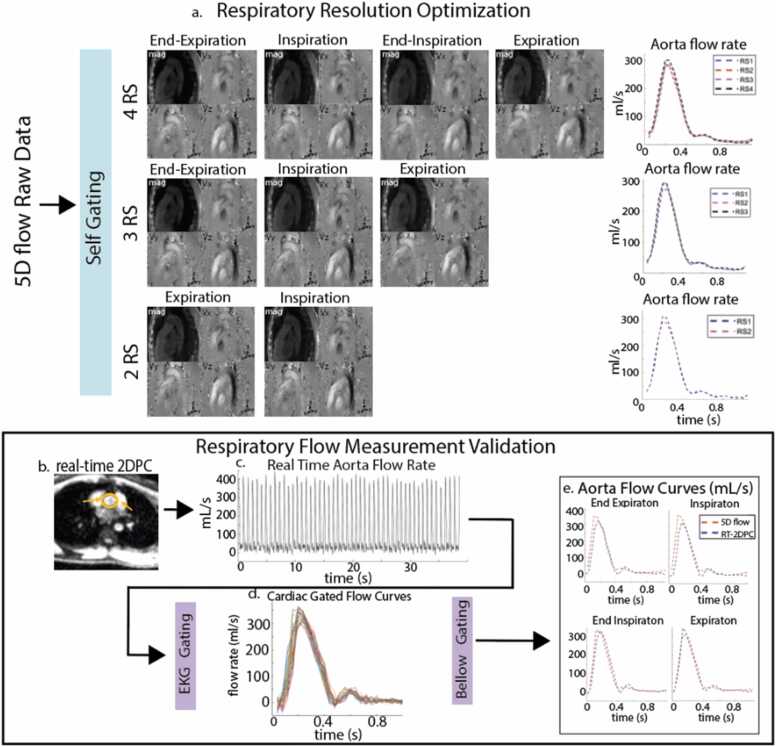
Reconstruction and analysis workflow. 5D flow CMR data were reconstructed with three different respiratory state resolutions using self-gating (a) and were compared for respiratory resolution optimization. Real-time 2DPC CMR was analyzed using time-resolved regions of interest (b) to measure real-time flow curves (c) in each vessel. ECG-gating (d) and respiratory bellows signals were used to gate the flow curves for comparison with 5D flow CMR (e). *5D* five-dimensional, C*MR* cardiovascular magnetic resonance , *2DPC* two-dimensional phase contrast, *ECG* electrocardiogram, *RS* respiratory state.

All RT-2DPC CMR images were reconstructed on the scanner and were analyzed using commercial software (Circle 5.14.2 Cardiovascular), including preprocessing with eddy current correction and velocity anti-aliasing. Each vessel lumen (AAo, MPA, IVC, and SVC) was manually delineated for each time frame (Fig. 2b). The resulting real-time flow curve measurements (Fig. 2c) were then gated using EKG and respiratory bellows. ECG signal was used for cardiac gating (temporal resolution = 37.5 ms, Fig. 2d). Respiratory bellows signal was bandpass filtered based on hypoventilation (6 breaths/min) and hyperventilation (20 breaths/min) thresholds. The filtered signal was respiratory gated using the method described above for 5D flow CMR. Measurements within the same cardiac and respiratory bin were averaged. Due to heart rate variability captured by the real-time measurements, flow curves were truncated before cardiac and respiratory gating to have the same heartbeat duration as the flow curves measured from 5D flow CMR (Fig. 2e). Net flow and peak flow were then measured.

### 2.8 Analysis: respiratory-driven flow validation

For both 5D flow CMR and RT-2DPC CMR data, the relative flow change due to respiration was defined as the range of the net flow normalized to the mean of the net flow over all respiratory states (Eq. 1).

(1) $$\mathrm{Relative flow change}=\;\frac{\mathrm{range}(\mathrm{net flow})}{\mathrm{mean}(\mathrm{net flow})}\times 100\%\;$$

Additionally, respiratory-resolved net flow and peak flow curves were normalized to end-expiration. The median normalized net and peak flow curves for the cohort were calculated for RT-2DPC CMR and each reconstructed 5D flow CMR dataset.

### 2.9 Statistical analysis

For all comparisons, data were tested for normality using a Shapiro-Wilks test. If the data were not normally distributed, Wilcoxon rank sum tests were used in place of t-tests. Bonferroni correction was used for the correction of multiple comparisons.

The respiration-resolved median net flow and peak flow were compared between RT-2DPC CMR and 5D flow CMR reconstructed with four respiratory states. Relative flow changes over all four respiratory states were compared using Spearman correlation. Spearman correlation was used to assess agreement between temporally aligned respiratory bellows signal and respiratory self-gating signal.

To assess the impact of respiratory state resolution in the entire cohort of healthy volunteers, Bland-Altman and correlation analysis were used to compare the net flow changes with four and three as well as three and two respiratory states. The respiration-resolved median net and peak flow with two and three respiratory statess were compared with 5D flow CMR reconstructed with four respiratory states. Additionally, a sub-group analysis was performed in the CHD cohort. Relative net flow changes in the AAo, pulmonary arteries (MPA, LPA, and RPA), and caval veins (IVC and SVC) were compared between CHD groups (SVD and shunt patients, respectively) and within groups to assess the impact of respiratory resolution. The impact of respiration on Qp:Qs was assessed within each group. In addition, the influence of respiration on APC burden was assessed in patients with the Fontan connection. Correlation analysis was used to assess the relationship between respiratory-driven relative flow changes and shunt volume in shunt physiology patients. Finally, 5D flow CMR measured net flow and Qp:Qs measurements were compared with clinically derived measurements from 2D phase-contrast CMR as available in clinical reports.

## 3 Results

### 3.1 Respiratory signal comparison

In one case, the respiratory bellows signal was not properly recorded during the 5D flow CMR acquisition. In the remaining volunteers, a strong correlation between the respiratory bellows and 5D flow CMR-derived respiratory self-gating signals was observed (ρ = 0.71 ± 0.15, p < 0.001 for all subjects). The average time shift between the self-gating and bellows signals was 0.18 ± 0.4 s. A case with strong self-gating vs bellows correlation (ρ = 0.77) is shown in Supplemental Fig. 1a. Supplemental Fig. 1b illustrates a case with moderate correlation (ρ = 0.58). Notably, this case had two regions of shallow or irregular breathing, which were successfully captured by both self-gating and bellows recordings (red boxes, Supplemental Fig. 1b).

### 3.2 Validation of 5D flow CMR against RT-2DPC CMR

**Fig. 3:**
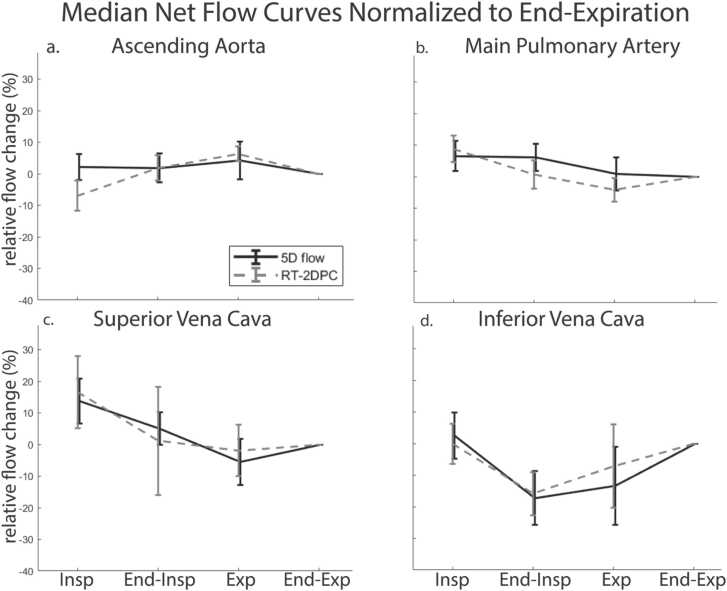
Median net flow curves normalized to end-expiration measured from 5D flow CMR and RT-2DPC CMR. Curves across all vessels show good agreement with significant differences. Expiratory increases are noted in the aorta (a), and inspiratory increases are noted in the MPA (b). *5D* five-dimensional, C*MR* cardiovascular magnetic resonance , *RT-2DPC* real-time two-dimensional phase-contrast , *MPA* main pulmonary artery.

**Fig. 4:**
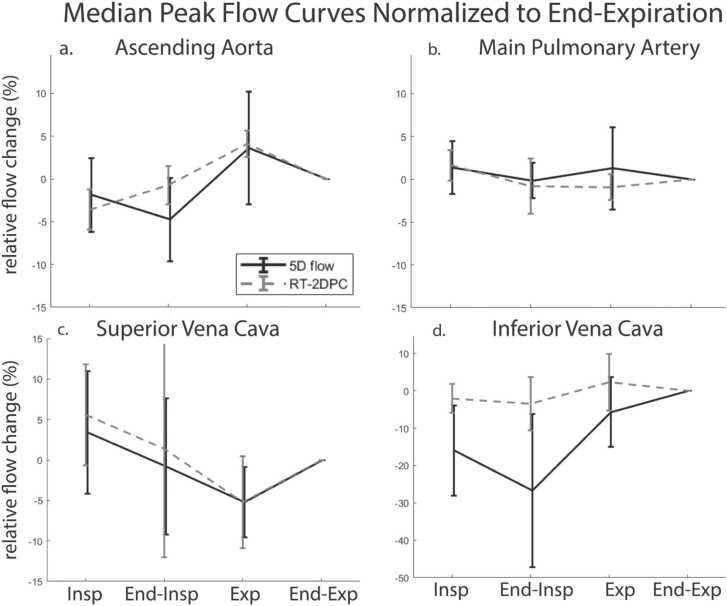
Median peak flow curves resolved over respiration for 5D flow CMR and RT-2DPC CMR. Curves across all vessels show good agreement with significant differences. The caval veins demonstrated the largest respiratory-driven peak flow changes. *5D* five-dimensional, *CMRI* cardiovascular magnetic resonance , *RT-2DPC* : real-time two-dimensional phase-contrast .

In the cohort of 10 volunteers, we found good agreement between the magnitude of respiratory-driven changes in net flow measured by 5D flow CMR and RT-2DPC CMR (Supplemental Fig. 2, ρ = 0.64, p < 0.001). Additionally, normalized, respiratory-resolved median net flow curves showed good agreement in all four analyzed vessels, with no significant differences (Fig. 3). Normalized respiratory-resolved peak flow curves also showed good agreement as well with no significant differences (Fig. 4).

### 3.3 Impact of respiration in healthy volunteers: 5D flow CMR

Respiration drove significant changes in net flow in several vessels when compared to end-expiration. The MPA had significant increases in net flow during inspiration (8.4%, p = 0.004, Fig. 3b) and end-inspiration (7.8%, p = 0.004, Fig. 3b). There was a significant decrease in net flow in the IVC during end-inspiration (−18.7%, p = 0.002, Fig. 3d). There were no significant differences in peak flow over the respiratory cycle, when compared to end-expiration.

### 3.4 Example case

**Fig. 5:**
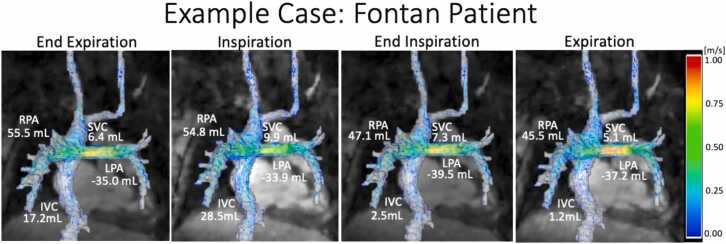
Example pathlines at 440 ms from a four respiratory state 5D flow CMR dataset collected in a Fontan patient. Net flows for each vessel are reported on the image. Note the substantial changes in IVC flow in each respiratory state. Time-resolved pathlines over the entire cardiac cycle are provided in Supplemental Video 1. *5D* five-dimensional, *CMR* cardiovascular magnetic resonance , *IVC* inferior vena cava, *RPA* right pulmonary artery, *SVC* superior vena cava, *LPA* left pulmonary artery.

In Fig. 5, example pathlines for four respiratory states during diastole (440 ms) are shown for an SVD patient status-post Fontan procedure (12 years, F). Notably, there were large changes in IVC net flow from inspiration to expiration with a 96% decrease in the net flow (28.8 vs 1.2 mL), indicating the importance of respiration-resolved flow imaging to capture flow changes in this patient group. Smaller changes were noted in the SVC and branch pulmonary arteries. In addition, time-resolved pathlines over the entire cardiac cycle demonstrated retrograde flow into the IVC from the conduit during expiration, likely contributing to the measured differences (Supplementary Video 1).

Supplementary material related to this article can be found online at doi:10.1016/j.jocmr.2024.101077.

The following is the Supplementary material related to this article Video 1.

Video 1

### 3.5 Impact of respiratory state resolution: CS acceleration

In healthy volunteers using four respiratory states, 19 ± 3% of k-space lines were gated into inspiration, 25 ± 2% into end-inspiration, 28 ± 2% into expiration, and 28 ± 2% into end-expiration (Table 2). Thus, inspiration and end-inspiration required the largest degrees of compressed sensing acceleration for 5D flow CMR image reconstruction (R = 46 ± 9 and R = 35 ± 6). This was also true for CHD patients (inspiration: R = 39 ± 9 and end-inspiration: R = 31 ± 7). These respiratory states were combined in the three RS reconstructions, significantly reducing the necessary compressed sensing acceleration factors (volunteers: R = 20 ± 3, p < 0.001; CHD: R = 17 ± 4, p < 0.001). The combination of the expiratory respiratory states for two RS reconstructions further reduced the necessary acceleration (volunteers: R = 15 ± 3, p = 0.02; CHD: R = 13 ± 3, p < 0.001). Table 2 summarizes acceleration factors for each respiratory state as well as the percent of measured k-space lines in each respiratory state.

**Table 2:** Acceleration factors for each respiratory state for both the volunteer and CHD cohorts.

Cohort	# Respiratory states	CS acceleration factor (R)			
		Inspiration	End-inspiration	Expiration	End-expiration
Volunteers	4	46 ± 9 (19 ± 3%)	35 ± 6 (25 ± 2%)	31 ± 6 (28 ± 2%)	31 ± 6 (28 ± 2%)
	3	**20 ± 3** (44 ± 3%)		31 ± 6 (28 ± 2%)	31 ± 6 (28 ± 2%)
	2	**20 ± 3** (44 ± 3%)		**15 ± 3** (46 ± 3%)	
CHD patients	4	39 ± 9 (19 ± 3%)	31 ± 7 (24 ± 1%)	26 ± 8 (28 ± 3%)	25 ± 5 (29 ± 2%)
	3	**17 ± 4** (42 ± 3%)		26 ± 8 (28 ± 3%)	25 ± 5 (29 ± 2%)
	2	**17 ± 4** (42 ± 3%)		**13 ± 3** (58 ± 3%)	

*CHD* congenital heart disease. *CS* compressed sensing

The percent of measured k-space in each respiratory bin is reported in parentheses. Bolded values indicate a significant reduction in compressed sensing acceleration factor R compared to the four RS reconstructions (p < 0.05). The acceleration factor is measured as the number of spokes necessary to fill the 96 × 96 × 96 matrix divided by the number of spokes acquired. This was averaged over the cardiac dimension.

### 3.6 Impact of respiratory state resolution: healthy volunteers

**Fig. 6:**
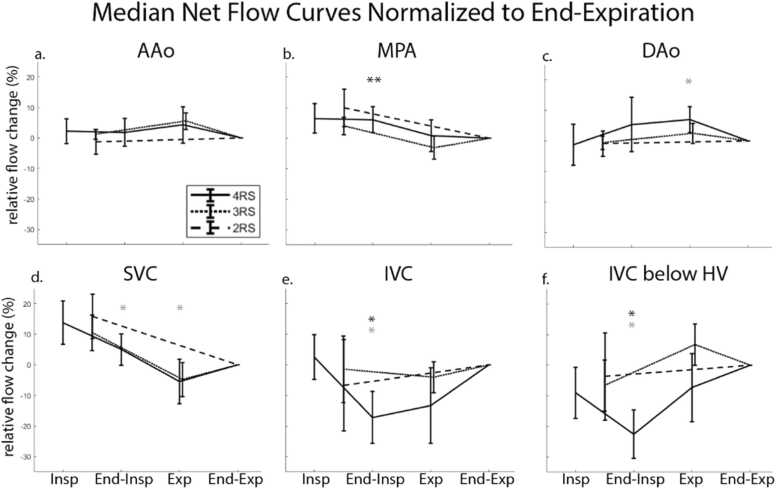
Median net flow curves normalized to end-expiration compare 5D flow CMR data reconstructed with two, three, or four respiratory states. Large variability between reconstructions was seen in the IVC above and below the hepatic veins. Gray asterisk indicates a difference between the two RS and four RS images. Black asterisk indicates a difference between the three RS and four RS images. *p < 0.05, **p < 0.01. *5D* five-dimensional, *CMR* cardiovascular magnetic resonance, *IVC* inferior vena cava, *AAo* ascending aorta, *MPA* main pulmonary artery, *SVC* superior vena cava, *DAo* descending aorta, *RS* respiratory state.

In several vessels, the four RS reconstructions had significantly different relative flow changes compared to the reduced respiratory resolution reconstructions (Fig. 6). Compared to the three RS images, the normalized net flow change was significantly different during end-inspiration in the MPA (6.1% vs 4%, p = 0.006, Fig. 6b), IVC (−17.2% vs −1.5%, p = 0.01, Fig. 6e), and IVC below the hepatic veins (−22.5% vs −6.6%, p = 0.01, Fig. 6f). The normalized peak flow was different in inspiration in the AAo (−1.9% vs 6.4%, p = 0.049) and in end-inspiration in the AAo (−4.8% vs 6.4%, p = 0.02), IVC (−26.7% vs −14.7%, p = 0.04), and IVC below the hepatic veins (−20.1% vs −5%, p = 0.01). Compared to the two RS images, the normalized net flow was different in end-inspiration in the SVC (5% vs 15.8%, p = 0.01, Fig. 6d), IVC (−17.2% vs −6.7, p = 0.02, Fig. 6e), and IVC below the hepatic veins (−22.5% vs −3.7, p = 0.03, Fig. 6f) and in expiration in the SVC (−5.5% vs 0%, p = 0.03, Fig. 6d) and DAo (7% vs 0%, p = 0.04, Fig. 6c). Peak flow was significantly different only in the IVC below the hepatic veins in end-inspiration (−20.1% vs −4.8%, p = 0.03).

**Fig. 7:**
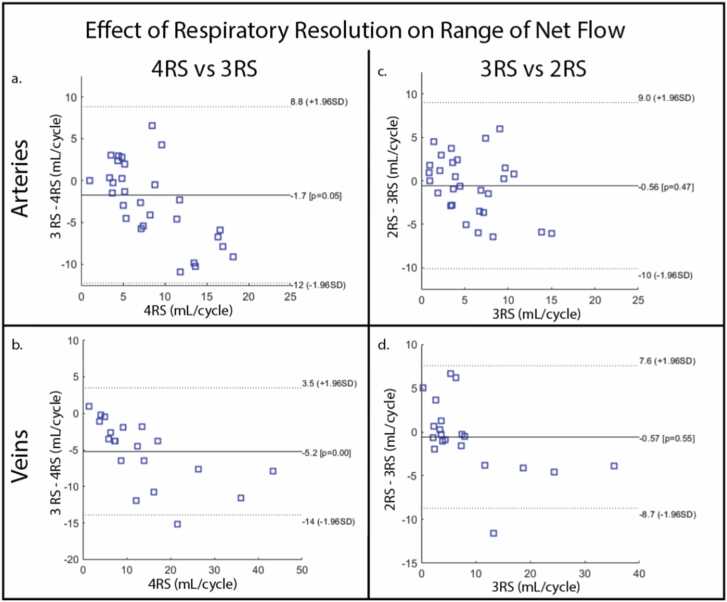
Bland-Altman plots demonstrating underestimation of the range of net flow measured over respiration in the three RS images compared to the four RS images in the arteries (a) and veins (b). However, there was a good correlation in the arteries (ρ = 0.61, p < 0.001) and veins (ρ = 0.76, p < 0.001). Bland-Altman plots demonstrate no bias in arterial (c) or venous (b) measurements in the three RS images compared to the two RS images. Good correlation was also observed in the arteries (ρ = 0.65, p < 0.001) and veins (ρ = 0.67, p = 0.002). RS, respiratory state.

The differences in respiratory-resolved net flow curves were also reflected in the range of flow measured over respiration as summarized by the Bland-Altman plots shown in Fig. 7. When comparing the arterial measurements in the four RS images to the three RS images, mild but significant underestimation of the respiratory-driven net flow changes was seen (1.7 mL/cycle, p = 0.05, Fig. 7a). More substantial underestimation was observed in the venous measurements (5.2 mL/cycle, p < 0.001, Fig. 7b). However, there was a good correlation in the relative net flow changes in both the arteries (ρ = 0.61, p < 0.001) and veins (ρ = 0.76, p < 0.001). When comparing the three RS images to the two RS images, no significant bias was found in either the arterial (Fig. 7c) or venous (Fig. 7d) net flow measurements. In this comparison, a good correlation was also observed from the relative net flow changes in the arteries (ρ = 0.65, p < 0.001) and veins (ρ = 0.67, p = 0.002).

### 3.7 Validation against clinically acquired 2D flow MRI: CHD patients

There was no difference in net flow for any vessel between 2D flow measurements and 5D flow measurements averaged over all respiratory states (p > 0.24). Average measured net flow and average differences between methods for each vessel can be found in Supplementary Table 1. The number of measurements available for comparison in each vessel is also reported in Supplementary Table 1.

Due to the limited availability of clinically acquired pulmonary venous flow measurements, Qp:Qs was compared in shunt patients only. The average Qp:Qs measured by 5D flow MRI was 1.74 and the average measured by 2D flow CMR was 1.73 (mean difference: 0.06, p = 0.6).

### 3.8 Impact of respiration on net flow: CHD patients

One single ventricle patient had a poorly visualized IVC in one respiratory state so measurement of this subject’s IVC was excluded. Over the entire CHD cohort, respiration had a significant impact on measured net flow when reconstructed with four respiratory states. During inspiration, the flows in the LPA (11 ± 19%, p = 0.003), SVC (15 ± 27%, p = 0.04), and IVC (24 ± 48%, p = 0.01) were significantly different from end-expiration. During end-inspiration, the MPA (6 ± 10%, p = 0.005), LPA (6 ± 37%, p = 0.049), and IVC (−11 ± 26%, p = 0.01) had significant flow changes relative to end-expiration. During expiration, only the IVC had a significant change in net flow from end-expiration (−26 ± 35%, p = 0.002). There were also differences in the respiratory-driven flow changes (Eq. 1) between the CHD subgroups. The SVD patients had greater respiratory-driven flow changes in both branch pulmonary arteries (RPA: 46% vs 15%, p = 0.003; LPA: 59% vs 20%, p = 0.002) and the SVC (51% vs 29%, p = 0.04) compared to the shunt patients.

### 3.9 Impact of respiration on Qp:Qs and collateral flow quantification: CHD patients

Pulmonary vein measurements were not possible in one Fontan patient due to complex anatomy. SVD patients had a median Qp:Qs of 1.1 ± 0.83 with a median range over respiration of 0.39 ± 0.21. Maximum Qp:Qs was found during active inspiration in 4/7 patients. Minimum Qp:Qs was measured during active expiration in 4/7 patients. Shunt patients had a median Qp:Qs of 1.82 ± 1.39 with a median change over respiration of 0.22 ± 0.14. Maximum Qp:Qs was measured during active inspiration in 4/11 patients, end-inspiration in 4/11 patients, and end-expiration in 3/11 patients. Minimum Qp:Qs was measured during active expiration in 5/11 patients, end-expiration in 3/11 patients, and end-inspiration in 3/11 patients. There were no significant correlations between shunt volume averaged over respiration and relative changes in flow in any vessels in shunt physiology patients. However, there were trends in the IVC (r = 0.6, p = 0.06), SVC (r = 0.4, p = 0.26), and AAo (r = 0.4, p = 0.23).

Fontan patients had a median APC burden of 16.2 ± 25.4 mL/cycle. The median relative change in APC burden over respiration was 118 ± 84%. In all patients, the minimum APC burden was measured during active inspiration. In 4/5 patients, the maximum APC burden was measured during active expiration (the remaining patient had maximal APC burden during end-inspiration).

### 3.10 Impact of RS resolution: CHD patients

**Fig. 8:**
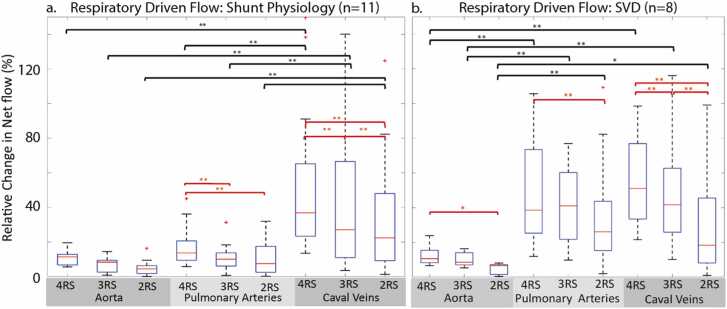
Impact of respiratory resolution in shunt physiology patients (a) and SVD patients (b). The caval veins were sensitive to all changes in respiratory state resolution in both patient groups. Black asterisks indicate a difference between vessels and red asterisks indicate differences between respiratory state resolutions. Relative change in net flow defined by Eq. (1). *SVD* single ventricle disease , *RS* respiratory state.

Similar to the healthy volunteers, reduced respiratory resolution had a significant impact on the degree of measured respiratory-driven flow changes (Fig. 8). In the shunt patients, the relative flow changes in the pulmonary arteries were decreased in the three RS images (16.5% vs 10.5%, p < 0.001) and two RS (16.5% vs 10%, p < 0.001) images compared to the four RS images. In the caval veins, there was a decrease in measured net flow change with each reduced respiratory state (Figs. 8a, four RS: 56.5%, three RS: 45.2%, two RS: 31.1%, p < 0.005 for all comparisons). However, regardless of respiratory state resolution, the caval veins had significantly more respiratory-driven flow changes than the pulmonary arteries and AAo (Fig. 8b). In SVD patients, a reduction from four to two respiratory states resulted in significantly less detected respiratory-driven flow in the AAo (12.4% vs 4.7%, p = 0.02) and pulmonary arteries (50% vs 34%, p = 0.005). A significant reduction in measured flow changes with each reduced RS was also found in the caval veins (Fig. 8a four RS: 63.9%, three RS: 45.4%, two RS: 31.0%, p ≤ 0.005 for all comparisons). Within all three reconstructed image sets, the AAo demonstrated less respiratory-driven flow than the pulmonary arteries and the caval veins (Fig. 8b).

## 4 Discussion

The findings of our study demonstrated the feasibility of 5D flow CMR for assessing respiration-resolved 3D blood flow dynamics in patients with CHD. Our main study findings are as follows. (1) There is strong agreement between the self-gating respiratory signal and respiratory bellows signal which served as the reference standard. (2) There is good agreement in respiratory-resolved net flow and peak flow changes between 5D flow CMR and RT-2DPC CMR. (3) Respiration has a substantial impact on net flow in the pulmonary arteries and caval veins in patients with shunt and single ventricle physiology. (4) Respiration had a substantial impact on measured Qp:Qs in SVD patients and moderate impact in shunt patients. (5) APC burden has substantial variation over respiration with the highest burden measured during active expiration in Fontan patients. (6) Venous flow measurements require a minimum of four respiratory states for complete evaluation of respiratory-driven flow changes, but fewer may be necessary for arterial flow measurements.

The results of the validation study in healthy volunteers demonstrated strong agreement between the respiratory bellows signal and the 5D flow CMR-derived respiratory signal. Similar to prior studies, there was a shift in the timing of peak inspiration and peak expiration [22]. However, good agreement in respiratory-resolved measurements was still found. In addition, relative changes in net flow and peak flow over the respiratory cycle were comparable between 5D flow CMR and RT-2DPC CMR. The remaining differences in the respiration-resolved median flow curves may be explained by acquisition method differences and manual segmentation variability. First, we used respiratory bellows and ECG leads for retrospective gating of the RT-2DPC CMR flow measurements as we could not replicate the self-gating inherent to 5D flow MRI. Second, the imaging slab for RT-2DPC CMR was static, and the vessels of interest moved with respiration throughout the acquisition. This may have been particularly impactful for the IVC, where the hepatic vein anastomosis may enter and exit the imaging slab over the acquisition. Additionally, the RT-2DPC CMR method used a Cartesian EPI acquisition with kt-generalized autocalibrating partially parallel aquisitions accelerated reconstruction whereas 5D flow CMR was radially acquired and reconstructed using compressed sensing. 5D flow CMR may thus be subject to more temporal blurring than the RT-2DPC CMR method. Further, RT-2DPC CMR was acquired with a longer repetion time and decreased spatial resolution compared to 5D flow CMR. The increased repetion time may have blunted measured peak flows, and the decreased spatial resolution may have driven errors due to partial volume effects. Lastly, the 5D flow CMR measurement locations were manually matched with the RT-2DPC CMR-acquired plane. This inevitably leads to slight variations in measurement location between the two methods. While this is a novel validation of respiratory-resolved 5D flow CMR, our results agree with prior studies. For instance, we showed that aortic net flow tends to increase during expiration and MPA net flow significantly increases during inspiration, which is in agreement with prior invasive measurements [12], [14].

In healthy volunteers, we found a significant impact of respiration on net flow in the IVC and MPA. These results suggest future work should investigate the impact of respiration on other hemodynamic metrics, such as kinetic energy, and that respiratory state during image acquisition must be considered in the longitudinal assessment of patients using phase-contrast CMR.

In CHD patients, we found good agreement between 5D flow CMR and clinically acquired 2D flow CMR net flow and Qp:Qs measurements. Furthermore, our study showed that respiration had a substantial impact on the net flow measured in both shunt and SVD patients, and that the impact of respiration on a vessel was anatomy dependent. Respiration had a significantly greater impact on pulmonary arteries in SVD patients than in shunt patients. This was expected as the pulmonary arteries of SVD patients are filled passively by the systemic venous return [23], while the pulmonary arteries are filled actively by right ventricular contraction in shunt patients. Additionally, it was plausible that respiration drove substantial changes in venous net flows. It has been previously shown that the passive pulmonary system of SVD patients has more respiratory-driven pulsatility than cardiac-driven pulsatility [24]. Additionally, Bastkowski et al. found an average of 120% change in IVC net flow from expiration to inspiration in a cohort of patients with the Fontan connection [11]. Another study using RT-2DPC CMR found a 73% respiratory-driven variation in IVC net flow in Fontan patients compared to 22% variation in healthy volunteers [13]. Our study also demonstrated substantial flow changes in the caval veins of SVD patients with smaller changes in the healthy volunteers. We also show significant differences in relative flow changes across the respiratory cycle in the IVC, highlighting the importance of consistent respiratory control in studies of CHD patients. Moreover, the degree of change over respiration measured by 5D flow CMR could be expected to change clinical reporting.

We also aimed to investigate the impact of respiration on Qp:Qs and shunt volume, metrics used in the clinical setting [17], [18]. In SVD patients, we found that the median change in Qp:Qs over respiration was approximately 37% of average Qp:Qs over respiration. Additionally, we found APC burden, a measure associated with adverse clinical outcomes in Fontan patients [25], had substantial variability over respiration. Importantly, we showed that peak changes in APC burden were measured during active inspiration and active expiration, respiratory phases not captured by conventional flow imaging. This suggests that optimal measurement of APC burden may not be possible with current clinical methods and that 5D flow CMR has the potential to impact clinical reporting.

In shunt patients, there was a smaller degree of variation in Qp:Qs over respiration, approximately 12% change over respiration. Qp:Qs tended to be smallest during expiratory phases and increased during inspiratory phases, highlighting the need for consistent respiratory control in serial evaluations of shunt patients. There was also a nonsignificant trend suggesting increased shunt volume may be associated with increased impact of respiration on IVC flows. This association may have been underpowered or obscured by the heterogeneity of shunt types included and should be investigated in larger cohorts.

Reducing the number of respiratory states reduces acceleration factors necessary for the compressed sensing image reconstruction by reducing radial undersampling. However, our results also showed that the reduction by a single respiratory state from four to three led to a significant reduction in information measured in the caval veins, even in healthy volunteers and more pronounced in CHD patients. However, arterial net and peak flow measures were robust to the reduction of respiratory states. Studies focusing on arterial flow measures may thus be feasible with a reduced number of respiratory states. This can either reduce the acceleration factor of these large datasets, while maintaining total 5D flow CMR scan time, or reduce scan time, while maintaining the degree of compressed sensing acceleration. A notable difference in CHD patients compared to healthy volunteers was found in shunt patients, where the pulmonary arteries were also sensitive to a reduction in the number of respiratory states. Our results suggest that at least four respiratory states should be used to evaluate respiratory-resolved flow changes in caval veins in SVD and shunt patients. This agrees with a prior study showing the impact of respiratory state resolution using RT-2DPC CMR in a cohort of Fontan patients [24].

## 5 Limitations

The study had several limitations. First, there is no gold standard for measuring respiratory-resolved hemodynamic changes. While we were able to use RT-2DPC CMR as a reference measurement, there are several potential sources of error when comparing to 5D flow CMR as discussed above. Additionally, this study did not investigate the impact of contrast on flow measurements [26], which was used in the patient cohort. There was a qualitative improvement in image quality with contrast, aiding in vessel delineation, but the impact of this was not systematically quantified. Our study also did not address the potential impact of body or voxel size. Some CHD patient scans were acquired with a smaller voxel size than those used in adult controls, which may have detrimentally impacted the signal-to-noise ratio.

Additionally, our validation study was performed in adult subjects, whereas our target patient population was CHD patients. However, comparison of 5D flow CMR with clinically acquired 2D flow CMR demonstrated good agreement, suggesting the accuracy of 5D flow in the CHD population as well. Future work is necessary to validate respiratory-resolved measurements in CHD patients.

Another limitation of this study was restricting the maximum number of RSs to four. It is possible that increasing the number of RSs may be necessary to fully capture the respiratory-driven hemodynamics. It has been previously shown in Fontan patients using RT-2DPC CMR that four RSs would likely be insufficient to successfully capture all respiratory-related changes. Gabbert et al. proposed at least 20 respiratory phases are necessary to fully capture the effect of respiration on flow curves [24]. However, to maintain tolerable 5D flow CMR scan time, acceleration factors, and reconstruction power, we chose to not investigate increasing the number of RSs. Further studies should consider longer scan times to enable this investigation.

While we propose that reduced respiratory resolution may improve images through reduced compressed sensing acceleration factors, this study did not investigate the impact of directly changing the acceleration factor. It is possible that the degree of acceleration impacts measured flow changes independent of the respiratory gating strategy. However, in the CHD cohort, paired comparisons between vessels showed that relative differences in respiratory-driven flow changes were maintained irrespective of the respiratory resolution. This would not be expected if acceleration was the primary driver of the measured flow changes. Additionally, it is possible that motion within each respiratory bin, dependent on respiratory depth, and regularization across respiration may introduce differences in measured flow. The impact of both types of motion artifact has been investigated in anatomical data, [27], [28] but future studies are necessary to understand the impact of this motion on respiratory-resolved flow. However, if motion noise was driving the measured differences, we would not expect the paired comparisons between vessels in CHD patients to be maintained irrespective of the respiratory resolution.

There are several other limitations, including the lack of scan-scan repeatability, selection of the venc sensitivity, and the mixed use of general anesthesia in the CHD cohort. The experiment also included several breath-held scans before the 5D flow acquisition, which may impact the respiratory patterns of the subjects. Scan-scan reproducibility is necessary to establish the reliability of the flow-resolved measurements. Additionally, high-velocity noise, due to relatively high venc selection, may limit the ability to detect respiratory-driven differences in the caval veins. Lastly, 6 out of 19 CHD patients had general anesthesia during their clinically indicated CMR studies, and thus also their 5D flow CMR studies. It is well established that general anesthesia, which requires manual ventilation, can impact hemodynamic measurements [29], [30]. Future studies should investigate the impact of general anesthesia on 5D flow CMR-derived measurements.

Lastly, this study is limited to simple hemodynamic metrics. More complex hemodynamic measurements, such as viscous energy loss [31], [32] or stasis [33], [34], may have different sensitivities to respiratory resolution than luminal net flow and peak flow.

## 6 Conclusions

Respiratory-resolved 5D flow CMR measures of net flow and peak flow show good agreement with respiratory-gated RT-2DPC CMR. Our results suggest that venous measurements are highly sensitive to the number of respiratory states, but arterial measures may be more robust. Additionally, we show that respiration has a substantial impact on flow in the pulmonary arteries and caval veins of both SVD and shunt patients as well as APC burden in Fontan patients. Future work should assess the potential clinical role of respiratory-resolved hemodynamic measurements.

## Funding

NIBIB T32EB025766; NHLBI F30HL165805; NHLBI R01HL115828-09; 10.13039/501100001711SNSF Grant 320030B_201292 (M.S., C.R., and M.F.); NHLBI F31HL165915.

## Author contributions

**Cynthia K. Rigsby:** Writing – review and editing, Supervision, Project administration, Methodology, Data curation, Conceptualization. **Justin Baraboo:** Writing – review and editing, Visualization, Methodology, Funding acquisition, Data curation, Conceptualization. **Elizabeth Weiss:** Writing – review and editing, Writing – original draft, Visualization, Validation, Software, Project administration, Methodology, Investigation, Funding acquisition, Formal analysis, Data curation, Conceptualization. **Michael Markl:** Writing – review and editing, Supervision, Resources, Project administration, Methodology, Funding acquisition, Data curation, Conceptualization. **Matthias Stuber:** Writing – review and editing, Supervision, Methodology, Conceptualization. **Christopher W. Roy:** Writing – review and editing, Methodology, Conceptualization. **Mariana B.L. Falcao:** Writing – review and editing, Methodology, Conceptualization. **Liliana Ma:** Writing – review and editing, Funding acquisition, Conceptualization. **Joshua D. Robinson:** Writing – review and editing, Methodology, Data curation, Conceptualization.

## Ethics approval and consent

This study uses data collected by protocols approved by Northwestern University IRB and Ann & Robert H. Lurie Children’s Hospital IRB as outlined in the manuscript methods.

## Consent for publication

Not applicable.

## Declaration of competing interests

Elizabeth Weiss, Justin Baraboo, and Michael Markl report financial support was provided by the National Heart Lung and Blood Institute. The other authors declare that they have no known competing financial interests or personal relationships that could have appeared to influence the work reported in this paper.

## References

[1] Dyverfeldt P., Bissell M., Barker A.J., Bolger A.F., Carlhall C.J., Ebbers T. (2015). 4D flow cardiovascular magnetic resonance consensus statement. J Cardiovasc Magn Reson.

[2] Soulat G., Scott M.B., Allen B.D., Avery R., Bonow R.O., Malaisrie S.C. (2021). Association of regional wall shear stress and progressive ascending aorta dilation in bicuspid aortic valve. JACC Cardiovasc Imaging.

[3] Allen B.D., van Ooij P., Barker A.J., Carr M., Gabbour M., Schnell S. (2015). Thoracic aorta 3D hemodynamics in pediatric and young adult patients with bicuspid aortic valve. J Magn Reson Imaging.

[4] Jarvis K., Schnell S., Barker A.J., Garcia J., Lorenz R., Rose M. (2016). Evaluation of blood flow distribution asymmetry and vascular geometry in patients with Fontan circulation using 4-D flow MRI. Pediatr Radiol.

[5] Lawley C.M., Broadhouse K.M., Callaghan F.M., Winlaw D.S., Figtree G.A., Grieve S.M. (2018). 4D flow magnetic resonance imaging: role in pediatric congenital heart disease. Asian Cardiovasc Thorac Ann.

[6] Stankovic Z., Allen B.D., Garcia J., Jarvis K.B., Markl M. (2014). 4D flow imaging with MRI. Cardiovasc Diagn Ther.

[7] Markl M., Schnell S., Barker A.J. (2014). 4D flow imaging: current status to future clinical applications. Curr Cardiol Rep.

[8] Ma L.E., Yerly J., Piccini D., Di Sopra L., Roy C.W., Carr J.C. (2020). 5D flow MRI: a fully self-gated, free-running framework for cardiac and respiratory motion-resolved 3D hemodynamics. Radiol Cardiothorac Imaging.

[9] Walheim J., Dillinger H., Kozerke S. (2019). Multipoint 5D flow cardiovascular magnetic resonance - accelerated cardiac- and respiratory-motion resolved mapping of mean and turbulent velocities. J Cardiovasc Magn Reson.

[10] Falcao M.B.L., Di Sopra L., Ma L., Bacher M., Yerly J., Speier P. (2021). Pilot tone navigation for respiratory and cardiac motion-resolved free-running 5D flow MRI. Magn Reson Med.

[11] Bastkowski R., Bindermann R., Brockmeier K., Weiss K., Maintz D., Giese D. (2019). Respiration dependency of caval blood flow in patients with Fontan circulation: quantification using 5D flow MRI. Radio Cardiothorac Imaging.

[12] Wise R.A., Robotham J.L., Summer W.R. (1981). Effects of spontaneous ventilation on the circulation. Lung.

[13] Korperich H., Barth P., Gieseke J., Muller K., Burchert W., Esdorn H. (2015). Impact of respiration on stroke volumes in paediatric controls and in patients after Fontan procedure assessed by MR real-time phase-velocity mapping. Eur Heart J Cardiovasc Imaging.

[14] Olsen C.O., Tyson G.S., Maier G.W., Davis J.W., Rankin J.S. (1985). Diminished stroke volume during inspiration: a reverse thoracic pump. Circulation.

[15] Woods T.D., Patel A. (2006). A critical review of patent foramen ovale detection using saline contrast echocardiography: when bubbles lie. J Am Soc Echocardiogr.

[16] Yamasaki Y., Kawanami S., Kamitani T., Sagiyama K., Sakamoto I., Hiasa K.I. (2018). Noninvasive quantification of left-to-right shunt by phase contrast magnetic resonance imaging in secundum atrial septal defect: the effects of breath holding and comparison with invasive oximetry. Int J Cardiovasc Imaging.

[17] Webb G., Gatzoulis M.A. (2006). Atrial septal defects in the adult: recent progress and overview. Circulation.

[18] Rao P.S., Harris A.D. (2018). Recent advances in managing septal defects: ventricular septal defects and atrioventricular septal defects. F1000Res.

[19] Traber J., Wurche L., Dieringer M.A., Utz W., von Knobelsdorff-Brenkenhoff F., Greiser A. (2016). Real-time phase contrast magnetic resonance imaging for assessment of haemodynamics: from phantom to patients. Eur Radiol.

[20] Di Sopra L., Piccini D., Coppo S., Stuber M., Yerly J. (2019). An automated approach to fully self-gated free-running cardiac and respiratory motion-resolved 5D whole-heart MRI. Magn Reson Med.

[21] Weiss E, Baraboo J, Ma L, Falcão MBL, Roy CW, Robinson JD, et al. Respiration-resolved 5D flow MRI: impact of the number of respiratory states of blood flow quantification in congenital heart disease patients. International Society for Magnetic Resonance in Medicine Annual Meeting; 2023.

[22] Liu J., Spincemaille P., Codella N.C., Nguyen T.D., Prince M.R., Wang Y. (2010). Respiratory and cardiac self-gated free-breathing cardiac CINE imaging with multiecho 3D hybrid radial SSFP acquisition. Magn Reson Med.

[23] Wei Z., Whitehead K.K., Khiabani R.H., Tree M., Tang E., Paridon S.M. (2016). Respiratory effects on Fontan circulation during rest and exercise using real-time cardiac magnetic resonance imaging. Ann Thorac Surg.

[24] Gabbert D.D., Hart C., Jerosch-Herold M., Wegner P., Salehi Ravesh M., Voges I. (2019). Heart beat but not respiration is the main driving force of the systemic venous return in the Fontan circulation. Sci Rep.

[25] Pisesky A., Reichert M.J.E., de Lange C., Seed M., Yoo S.J., Lam C.Z. (2021). Adverse fibrosis remodeling and aortopulmonary collateral flow are associated with poor Fontan outcomes. J Cardiovasc Magn Reson.

[26] Kollar S.E., Udine M.L., Mandell J.G., Cross R.R., Loke Y.H., Olivieri L.J. (2022). Impact of ferumoxytol vs gadolinium on 4D flow cardiovascular magnetic resonance measurements in small children with congenital heart disease. J Cardiovasc Magn Reson.

[27] Roy C.W., Heerfordt J., Piccini D., Rossi G., Pavon A.G., Schwitter J. (2021). Motion compensated whole-heart coronary cardiovascular magnetic resonance angiography using focused navigation (fNAV). J Cardiovasc Magn Reson.

[28] Roy CW, Milani B, Yerly J, Si-Mohamed S, Tenisch E, Rutz T, et al. Intra-bin correction and inter-bin compensation of respiratory motion in free-running 5D whole-heart MRI. International Society for Magnetic Resonance in Medicine Annual Meeting; 2023. 10.1016/j.jocmr.2024.101037 PMC10987330 38499269

[29] Mesquida J., Kim H.K., Pinsky M.R. (2011). Effect of tidal volume, intrathoracic pressure, and cardiac contractility on variations in pulse pressure, stroke volume, and intrathoracic blood volume. Intensive Care Med.

[30] Kim H.K., Pinsky M.R. (2008). Effect of tidal volume, sampling duration, and cardiac contractility on pulse pressure and stroke volume variation during positive-pressure ventilation. Crit Care Med.

[31] Weiss E.K., Robinson J.D., Sodhi A., Markl M., Rigsby C.K. (2023). Impact of pulmonary artery flow distribution on Fontan hemodynamics and flow energetics. Pediatr Radiol.

[32] Kamphuis V.P., Roest A.A.W., van den Boogaard P.J., Kroft L.J.M., Lamb H.J., Helbing W.A. (2021). Hemodynamic interplay of vorticity, viscous energy loss, and kinetic energy from 4D Flow MRI and link to cardiac function in healthy subjects and Fontan patients. Am J Physiol Heart Circ Physiol.

[33] Geeraert P., Jamalidinan F., Burns F., Jarvis K., Bristow M.S., Lydell C. (2021). Hemodynamic assessment in bicuspid aortic valve disease and aortic dilation: new insights from voxel-by-voxel analysis of reverse flow, stasis, and energetics. Front Bioeng Biotechnol.

[34] Chu S., Kilinc O., Pradella M., Weiss E., Baraboo J., Maroun A. (2022). Baseline 4D flow-derived in vivo hemodynamic parameters stratify descending aortic dissection patients with enlarging aortas. Front Cardiovasc Med.

