# Supplementary material for: Respiratory-resolved five-dimensional flow cardiovascular magnetic resonance : In-vivo validation and respiratory-dependent flow changes in healthy volunteers and patients with congenital heart disease
Source: J Cardiovasc Magn Reson. 2024 Aug 2;26(2):101077. doi: 10.1016/j.jocmr.2024.101077 (PMC11417305; doi:10.1016/j.jocmr.2024.101077)
Supplement: Supplementary file 2 — Supplementary material [file mmc1.docx]

Supplemental Info

Supplemental Video 1. Pathlines from a 4 respiratory state 5D flow MRI data set collected in a Fontan patient.


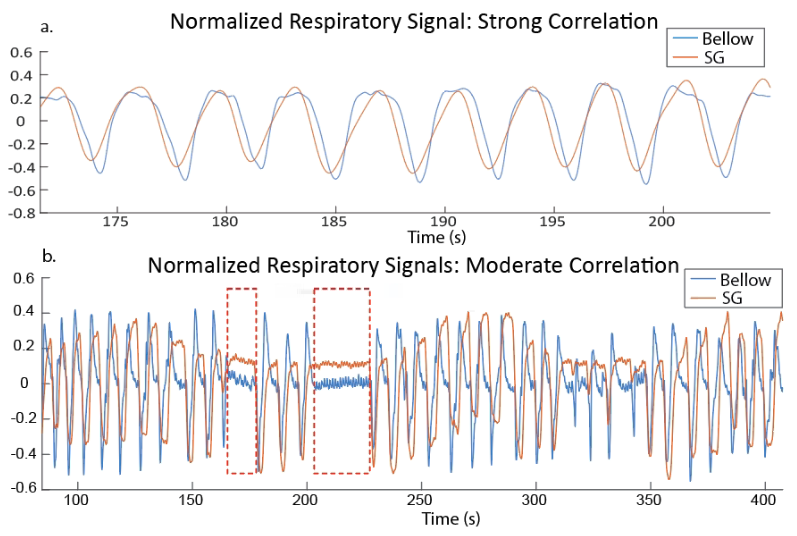


Supplemental Figure 1. A magnified portion in a case with strong agreement (ρ = 0.71) between the respiratory bellows and self-gating signal is shown (a). A case with moderate agreement (ρ = 0.58) is shown in b. The red boxes highlight regions of atypical respiratory signal captured by both modalities.


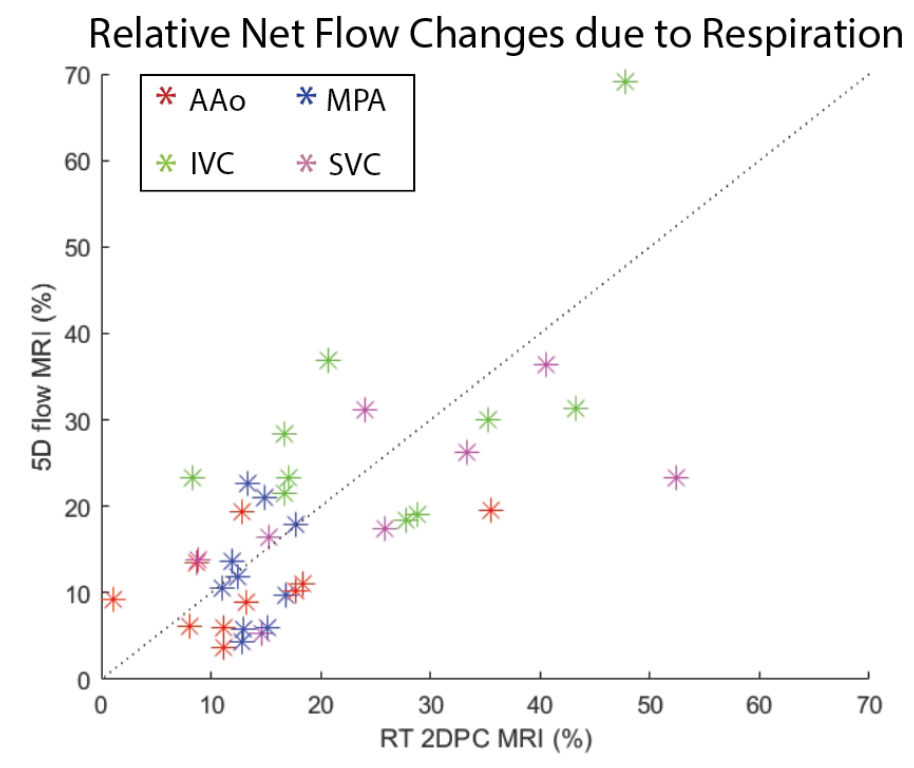


Supplemental Figure 2. Correlation in the relative, respiratory driven changes in net flow measures by 5D flow MRI vs RT-2DPC MRI (Eq 1). Correlation for each individual vessel is as follows: AV (ρ = .48, p = 0.17), MPA (ρ = 0.14, p = 0.7), IVC (ρ = 0.43, p = 0.22), SVC (ρ = 0.68, p = 0.04).

|  | **5D flow MRI**  **net flow (mL/cycle)** | **2D flow MRI**  **net flow (mL/cycle)** | **Paired**  **difference (mL/cycle)** |
| --- | --- | --- | --- |
| **Aorta** (18) | 54.7 | 51 | 3.3 (p=0.2) |
| **MPA** (12) | 72.7 | 66.8 | 4.1 (p=0.6) |
| **LPA** (14) | 23.6 | 20.3 | 1.3 (p=0.3) |
| **RPA** (12) | 34.4 | 32.5 | -4.3 (p=0.8) |
| **SVC** (6) | 19.5 | 11.4 | -0.6 (p=0.7) |
| **IVC** (7) | 33.4 | 28.0 | -2.8 (p=0.6) |

Supplemental Table 1. Average net flow measured by 5D flow MRI and 2D flow MRI in CHD patients. Note, not all vessels were available for comparison, number of patients per vessel is given in parenthesis.
